# Medical microrobots in reproductive medicine from the bench to the clinic

**DOI:** 10.1038/s41467-023-36215-7

**Published:** 2023-02-09

**Authors:** Richard Nauber, Sandhya R. Goudu, Maren Goeckenjan, Martin Bornhäuser, Carla Ribeiro, Mariana Medina-Sánchez

**Affiliations:** 1 Micro- and NanoBiomedical Engineering Group (MNBE) Institute for Integrative Nanosciences, Leibniz Institute for Solid State and Materials Research (IFW), 01069 Dresden, Germany; 2 Medical Clinic I, University Hospital, Technische Universität Dresden, Fetscherstraße 74, 01307 Dresden, Germany; 3 National Center for Tumor Diseases (NCT/UCC), Dresden, Germany; 4 Chair of Micro- and NanoSystems, Center for Molecular Bioengineering (B CUBE), Dresden University of Technology, 01062 Dresden, Germany

**Keywords:** Cell delivery, Health care, Biomedical engineering, Biomedical materials, Translational research

## Abstract

Medical microrobotics is an emerging field that aims at non-invasive diagnosis and therapy inside the human body through miniaturized sensors and actuators. Such microrobots can be tethered (e.g., smart microcatheters, microendoscopes) or untethered (e.g., cell-based drug delivery systems). Active motion and multiple functionalities, distinguishing microrobots from mere passive carriers and conventional nanomedicines, can be achieved through external control with physical fields such as magnetism or ultrasound. Here we give an overview of the key challenges in the field of assisted reproduction and how these new technologies could, in the future, enable assisted fertilization in vivo and enhance embryo implantation. As a case study, we describe a potential intervention in the case of recurrent embryo implantation failure, which involves the non-invasive delivery of an early embryo back to the fertilization site using magnetically-controlled microrobots. As the embryo will be in contact with the secretory oviduct fluid, it can develop under natural conditions and in synchrony with the endometrium preparation. We discuss the potential microrobot designs, including a proper selection of materials and processes, envisioning their translation from bench to animal studies and human medicine. Finally, we highlight regulatory and ethical considerations for bringing this technology to the clinic.

## Abstract (web summary)

Microrobotics is an emerging technology with wide implications for medical fields, such as assisted reproduction. Here the authors show how the key challenges regarding materials, processes and engineering as well as ethical and regulatory implications can be addressed towards a clinical adoption.

## Case study on infertility with recurrent implantation failure (RIF)

Infertility is a problem that affects 48.5 million couples worldwide^1^. The possible causes of the female factor are ovulatory disorders, tubal dysfunction, endometriosis, uterine and/or cervical factors. The male factor is usually caused by poor sperm quality (e.g., low motility, abnormal morphology, or low count), decreasing the possibility of fertilizing the oocyte in vivo. Common existing infertility treatments include low-cost and minimally invasive hormonal stimulation and intrauterine insemination, in vitro fertilization (IVF), or intracytoplasmic sperm injection (ICSI), which are indicated if tubal infertility or severe male infertility are diagnosed. The application of these techniques has rapidly increased due to improved protocols and better gamete selection techniques, suggested by international guidelines^2^, reaching fertilization rates of about 95%^3^. However, implantation rates for ICSI and IVF are still between 17 to 21% (after day 3) and decline with the patient’s age^4^. These rates have been further improved in the last years, after prolonged embryo cultivation in vitro (up to day 5), reaching pregnancy rates of 42–47%^5^. However, the probability to get high-quality blastocysts is still low and relies on the need of retrieving a high number of oocytes with hormone stimulation, but even with advanced quality assessment techniques, using machine learning, the implantation rate per embryo with optimal quality is still not higher than 57,5%^6^.

These low pregnancy rates of transferred embryos obtained by IVF and ICSI might be caused by the stress the gametes are exposed to during their in vitro manipulation^7^. Lifestyle factors, diseases, uterine or endometrial abnormalities, or embryonic factors might also have an impact. Differences in IVF-laboratory protocols have also shown an influence on the success of each treatment^8^. Nevertheless, in most cases, no apparent explanation is found. For those medical problems, treatment of endometrial injury, change in the stimulation protocol, transfer of the embryo at the blastocyst stage, and/or assisted hatching have been shown to help^9^.

In particular, for recurrent embryo implantation failure, a promising method in the first years after the introduction of IVF^10^ was the intrafallopian transfer of gametes/zygotes (GIFT/ZIFT) by laparoscopy^11^. This technique was abandoned after the extracorporal fertilization with IVF and ICSI was improved and the culture conditions in modern IVF laboratories showed higher embryo formation rates. However, GIFT and ZIFT are still considered advantageous as they offer an appropriate physiological environment for fertilization and/or embryo development, and an optimal synchronization between embryonic and endometrial preparation. This procedure has shown for some cases of RIF, higher pregnancy rates^12^, but in a metastudy, including three cases of ZIFT, no evident improvement was observed in the live birth rate^13^. In general, it is known that the success of the technique relies on the surgeon’s expertise and the applied protocol which differ among IVF laboratories. The traditional method is also quite invasive, requires anesthesia, and may have adverse effects^14^, However, less invasive techniques of ZIFT as microrobotic ZIFT/GIFT may result in a better outcome.

The microrobotic carriers at a small scale could be an appealing option for RIF or other infertility problems, in which it could be beneficial to transport both gametes (oocyte and sperm cells), early embryos, with and/or without other therapeutical cargoes, to the physiological fertilization site to let the embryo development occur under natural conditions. Active embryo carriers could be a solution to the loss of functional embryos due to the fertilization and cultivation process under laboratory conditions which imitate the physiological conditions only in part.

The question of asynchronous endometrial receptivity on the day of embryo transfer as a reason for implantation failure is also extensively studied^15^. The microrobotic intratubal transfer of early gametes/embryos (also called µGIFT/µZIFT) could lead to synchrony with endometrium preparation. Moreover, in some cases of oncological patients with contraindications of using drug stimulation, for example, kidney diseases, liver, heart pathology, blood vessels, and oncological diseases that want to conceive, it is known that they cannot be stimulated with hormones and they will benefit from the natural cycle, where the availability of oocytes is very low. In those cases, it could be beneficial to transport both gametes (oocyte and sperm cells) to the fallopian tube. Likewise, the transport of early embryos is also a promising alternative, as the embryo can develop under physiological conditions and implant in synchrony with the endometrium preparation.

**Fig. 1:**
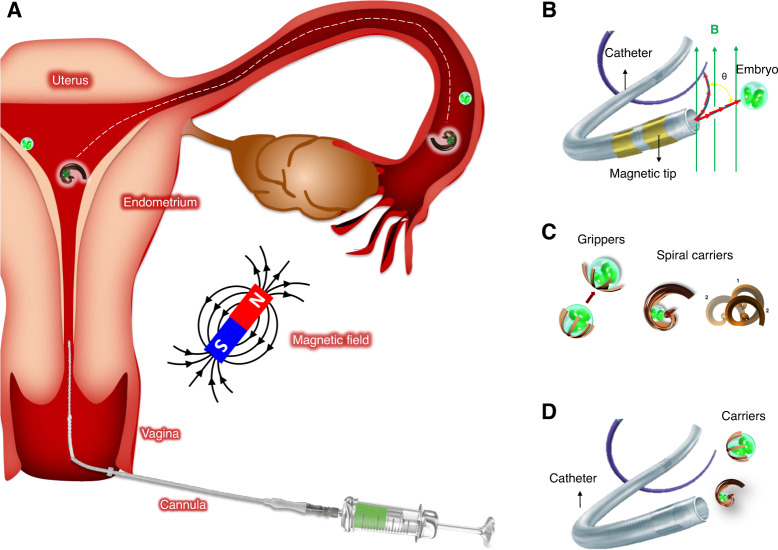
Case study—microrobotic embryo transfer or µZIFT. **A** Conceptual depiction of a spiral-like micromotor that is used to capture, transport, and release an oocyte or embryo in the fallopian tube and endometrium. Overview of strategies for embryo transfer: **B** Tethered approach using a microcatheter. **C** Untethered approach with microcarriers. **D** Combined approach deploying untethered carriers through a catheter.

Since the development of assisted reproduction with intrauterine embryo transfer, the method of transfer has not been modified. The procedure has a high intra- and interpersonal dependency^16^. Thus, we believe microrobotic tools (tethered or untethered) and methods to transfer non-invasively gametes or embryos back to the fallopian tube (narrow channels in the reproductive tract) are promising to increase pregnancy rates (Fig. 1)^17^. But to do so, these microrobots should have the ability to reliably capture and secure the gametes/embryo during transport through different environments, allow the access of the secreted molecules either by the oviduct ciliary cells or by the embryo, be biocompatible and/or biodegradable and not exceed the size of the minimum dimensions in the oviduct (ca. 500 µm), be able to move in viscoelastic media and against back flows in the fallopian tube (produced by peristaltic motion and cilia beating), and not harm the oviduct, which is a very delicate organ.

The first report towards µZIFT were spiral-like micromotors and helical micropropellers, the first one outperforming the established helical structure in terms of locomotion and ability to reliably capture and secure a large cargo during transport among different environments^18^. We demonstrated, in particular, the cargo-delivery of murine embryos in vitro, considering different microenvironments transfers and highly viscous media. These results were promising; however, further studies on embryo oxidative stress and the influence of the structure on the function of the fallopian tube and uterus remain to be studied.

Currently, our group is working on microrobots that can safely transport single embryos either to the endometrium or to the ampulla site of the fallopian tube, enabling embryo development under natural conditions in contrast to in vitro culture conditions, the same time as avoiding multiple pregnancies. For instance, a microcatheter tool containing active components for the diagnosis and release of healthy embryos, able to pass through the uterine cavity and the fallopian tube is here envisioned (see Fig. 1). Such a device has been partially demonstrated in previous work from our group, where rolled-up polymeric films were functionalized with electroactive polymers that serve as microactuators. The microcatheter devices were also integrated with sensors that provide information about the tip deformation and position, and functions such as fluid injection and cargo-delivery of microscopic cargoes were also successfully demonstrated^19^. Other groups have also developed similar concepts for operating mainly in vascular networks, using techniques like electrospinning or micromolding^20,21^. Larger catheters or flexible needles coated with magnetic materials or shape memory alloys, have been suggested as potential candidates for non-invasive medical operations with targeting capabilities^22–24^. On the other side, untethered microrobots with designs such as spirals, spherical microgrippers, or capsule-like embryo carriers are also promising alternatives (see Fig. 1). Such untethered microrobots can be transported and directed via magnetically controlled locomotion modes such as rolling, swimming, crawling, jumping, and walking, depending on their geometrical design. For example, grippers are one of the most commonly used robot types to transport and release cargo. The arms of the gripper can be deformed either magnetically^25^ or thermally^26^ to perform cargo grabbing and releasing for in vivo applications. When the gripper reaches its specific target location in a reproductive tract, the cargo can be released by tuning the applied stimuli. The gripper size should be comparable to the dimensions of the fallopian tube in the female reproductive system and the applied stimuli should not harm the embryos and the surrounding tissue. These microrobots can be fabricated by different methods (e.g., strain engineering, 3D/4D printing, electrospinning, etc.), in which soft and smart materials are patterned in the desired geometry^27^. Such materials can additionally be loaded/functionalized with antioxidants, hormones, and drugs, depending on the requirements, being soft and permeable for the exchange of nutrients from the secretory cells in the fallopian tube^28^.

The 3D manipulation of those microrobots in complex viscoelastic fluids and inside living organisms is also a major hurdle. To address this issue, we carried out preliminary tests to evaluate the feasibility of using high-frequency ultrasound (US) and photoacoustic imaging (PAI) to track in real-time magnetically actuated micro-objects which were in the same size scale as those we intend to use for this application (ca. 100 µm)^29^. This technique combines the advantages of ultrasound imaging (e.g., real-time, deep tissue), a resolution in the µm range, and near-infrared (NIR) molecular absorption which is beneficial to distinguish the spectral signatures of the micro-objects from the surrounding tissue molecules, being crucial for future in vivo studies. So far, we have achieved the visualization of such carrying structures in living mice, below ca. 1–2 cm, in real-time, enabling the initialization of preclinical trials in small animal models as a next step. To envision the translation of this technology to large mammalians and eventually to humans, we hereby discuss some considerations related to the materials, sterilization processes, and setups for their imaging and control. Finally, the ethical concerns and steps toward the successful approval of a clinical trial are put into perspective.

## Materials and processes for embryo-carrying microrobots

Ensuring the safety of medical microrobots has the highest priority when they are carrying out their medical tasks in vivo. Animal studies and use in veterinarian medicine can be envisioned and serve as a model for the possible use in human reproductive medicine^30^.

Once the specific medical task is achieved, the robots should be fully degraded or retrieved in the biological environment, ideally without additional surgery for removal. The degradation could be accomplished either enzymatically (facilitated by specific enzymes such as collagenase, matrix metalloproteinase (MMPs), which are present in the body) or by local pH and temperature changes. Thus, choosing the right material composition, including biocompatibility and biodegradability, is mandatory during the design process of a medical microrobot. Biodegradable materials, such as gelatin methacrylate (GelMA), collagen, silk, and alginate, provide sufficient mechanical support to the microrobot body. Young’s modulus of such biodegradable materials is in the order of kPa. Thus, they are very soft and adapt to the changes in the biological environment during movement toward the targeted location inside the human body. The robots’ flexibility and shape-changing ability allow them to actively pass through the biological barriers to access hard-to-reach anatomical locations in a minimally invasive manner. They also induce versatile shape deformations and undergo multimodal locomotion depending on the mechanical properties of the geometrical design, magnetization of the magnetic material, magnetization profile inside the robot body, applied external magnetic field, and the viscosity of the biological fluid. Tuning these properties to achieve highly flexible structures at the microscale becomes challenging since it requires precise predictions of finite deformations according to the desired medical application at the clinical level. Possible interactions of the microrobots, as well as the degradation products of the carrier with the organ surfaces, e.g., endometrium and the fallopian tube epithelial cells, with embryo development and implantation, should also be considered prior to transfer them to in vivo conditions. In case the sub-products of the degradation are not suitable for ensuring proper embryo development, the microrobot can be transported back to its initial position by an applied external magnetic field and be retrieved by a cannula.

Regarding the materials needed for their actuation, magnetic and ultrasound-driven microrobots seem to be the most promising ones for clinical applications because both magnetic and ultrasound-induced forces can efficiently and harmlessly penetrate through biological tissues. However, magnetic microrobots made of soft magnetic materials (Ni and Co thin films) are considered not biocompatible. Instead, metallic alloys such as FeMgSi or FePt are promising alternatives, some of them with the possibility to degrade within a few hours in the presence of biological-relevant fluids^31^. Further, embedding hard magnetic materials, including NdFeB, CrO_2_, and BaFe_12_O_19_, within the microrobot body is also considered toxic. According to the international standard ISO 10993.1 for the evaluation of medical devices^32^, not just the surface coatings of medical devices but the entire device should be biocompatible. In this regard, superparamagnetic iron oxide nanoparticles (SPIONs)^33^ and iron platinum (FePt) nanoparticles^34^ are categorized as bio-friendly and have shown significant advantages for medical use. Surface coatings with proteins^35^, DNA^36^, gold, and polymers such as parylene C^37^ and polyethylene glycol diacrylate (PEGDA)^38^ have also been used to improve microrobots’ biocompatibility. Furthermore, specific materials and coatings to avoid the micromotor stiction problem when operating in complex biological environments should be implemented. Employing cell camouflages, zwitterionic materials, enzymatic, or ferrite coatings might help, as has been demonstrated elsewhere^39–42^. In summary, both the morphology and surface chemistry of the microrobots should be optimized to minimize undesired physical interactions with the surrounding biological tissue and to avoid their rejection by the immune system^38^.

Processing of these materials is also of major relevance. Materials like hydrogels, and biodegradable polymers, both responding or not to stimuli such as temperature, pH, and electrical signals, require dedicated fabrication strategies. Methods like 2D or 3D lithography, micromolding, or microfluidic-mediated fabrication processes are promising for that purpose^43^. Currently, mass fabrication of such tiny medical robots towards tangible translation to market could not be achieved by any of the existing micro/nanofabrication technologies. Alternatively, bottom-up techniques, among which chemical synthesis and template-based electrodeposition have mass production potential and are commonly used for the synthesis of nanoparticles and micro/nanostructures. The suitability of the method is also closely related to the minimum feature size which can be produced, which is also a relevant factor, as the micromotor size will limit their application scenario and will enable/hinder their ability to penetrate different biological barriers/tissues, depending on the intended application.

Sterilization of these microrobots and in general any device meant to enter the human body is also critical. Sterilization methods typically involve the use of aggressive solvents, high temperatures, or exposure to UV lights for a certain period. Exposure to these steps might affect the integrity and function of the microrobots. Therefore, it is crucial to realize a proper selection of materials prior to their fabrication and sterilization. In particular, these carriers are mainly fabricated by polymers and soft materials, with just a few nanometers of inorganic layers (e.g., FePt and Au) for functions like magnetic actuation or imaging, respectively. Methods like exposure to ethylene oxide, radiation, dry heat and steam, hydrogen peroxide, and ozone might have a detrimental effect on those materials, particularly considering their small size. Then, one should evaluate novel and less harmful methods like peracetic acid exposure, UV light, microwaves, sound waves, or pulsed light^44^.

The abovementioned considerations are shared for all medical microrobots, but in particular, for embryo transport and release in the reproductive system, one should also consider the following: materials and actuation methods should not harm the embryo or the reproductive system. Regarding the material biocompatibility in relation to gametes and embryos, we have carried out preliminary studies in vitro in which no evident cytotoxicity or inflammatory response has been observed with our previously reported microcarriers^45–47^.

Moreover, they should be permeable to the nutrients/factors secreted by the fallopian tube and should ideally not remain in the body or close to the embryo during its development. Even in the case of successful embryo transport by one of these material systems, one should also evaluate factors like the embryo’s development, oxidative stress, and mutations, among other factors that might compromise the integrity and function of the  gametes/embryos and the reproductive tract.

## Actuation and imaging of microrobots in large mammals and eventually in humans

**Fig. 2:**
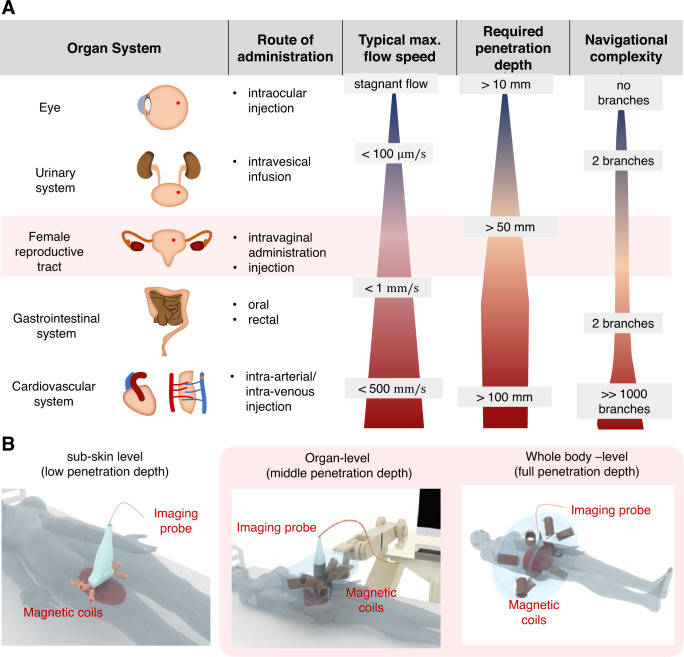
Overview of medical microrobotics targeting different organ systems. **A** Administration routes for alternative applications such as targeted drug delivery, microsurgery, local sensing, microbiopsy, and tissue engineering, highlighting the key challenges for operating such microrobots through these administration routes (e.g., required propulsion forces, required penetration depth for both imaging and actuation, and complexity of the environment in which the microrobots navigate in). **B** Scenarios of sub-skin, organ-level, and whole-body scale application of a combined magnetic actuation and ultrasound/photoacoustic imaging unit. Red shadowed boxes highlight the particular scenario for the treatment of diseases in the reproductive system as well as point out the most suitable imaging and actuation settings for noninvasive operations in it.

Medical microrobots have been demonstrated for a variety of noninvasive biomedical applications. However, most of these demonstrations have been carried out in vitro and under optical microscopy, being significantly different from the clinical practice. For a targeted application of microrobots in human patients, external support depending on the level of autonomy must be provided. While autonomous systems usually require only offline imaging for monitoring the efficacy of therapy, remote-controlled systems need real-time imaging for localizing the microrobots, controlled actuation for propelling them despite the viscosity and flow of the surrounding media, and high-level navigation for guiding them toward their target. Depending on the targeted system inside the human body, the requirements for the three main components differ (Fig. 2A): the external propulsion system must overcome the forces exerted on the microrobots, such as viscous drag and flow, where the latter is especially high in the cardiovascular system. Furthermore, the penetration depth of imaging and actuation needs to meet the targeted organ system (Fig. 2B). With increasing geometrically and topological complexity in systems such as the reproductive tract or the cardiovascular system, navigation becomes a crucial aspect of successful microsurgery.

While tethered tools can be easily retrieved after use, untethered systems should be safe to remain in the body or retrieved from the location of administration. However, for successful operation, their movement must be restricted to a region where actuation and imaging are effective. Organ systems with restricted lumens and stagnant flows, such as the eye, or low flow, such as the fallopian tube with less than 1 mm/s^48^, it is ensured intrinsically that the microrobots will not be lost towards the rest of the body. In systems with strong flows, such as the cardiovascular system with a velocity ranging from 0.5–500 mm/s^49^, a hybrid approach of tethered deployment through a catheter and a reduction of the flow with a balloon can ensure that the microrobots do not leave the region of actuation and imaging.

In vivo imaging of microrobots is challenging in general because of their small size and the scattering properties of the tissue. Suitable imaging modalities can be classified by the mechanisms of contrast that they use: either optical, magnetic, mechanical, or due to radioactive decay. The imaging approach defines the spatial resolution, the penetration depth as well as compatibility with clinical practice, which can be considered the most relevant properties for in vivo applications. A comprehensive overview of different techniques is given by ref. ^50^. For example, infrared (IR) imaging is appealing for ophthalmology and sub-skin interventions as the penetration depth of light in tissue is comparably small. For applications like small animal imaging, with cm penetration depth, other techniques should be employed, such as US and photoacoustics. We showed for the first time real-time tracking of single moving micro-objects below cm thick phantom tissue and ex vivo chicken breast, using PAI^51^. The resulting PA signal was further improved in terms of contrast and specificity by coating the micro-object surface with gold nanorods. This coating possesses a unique absorption spectrum, which facilitates its discrimination from surrounding biological tissues when translated to future in vivo settings.

For operating at a human scale, imaging techniques like magnetic resonance imaging (MRI), nuclear techniques like positron emission tomography (PET), or single-photon emission computed tomography (SPECT) are established as diagnostic tools. However, their use in surgical procedures is hampered by the cost and clinical practicability as well as the exposure to ionizing radiation in the cases of PET and SPECT.

We predict that in the context of clinical applications, US-based modalities with contrast enhancement will play a central role in the real-time imaging of microrobots. US imaging, in general, can achieve high penetration depths in tissues while avoiding exposure to ionizing radiation. This, as well as its widespread clinical acceptance, cost efficiency, and flexibility, make it a great tool for microsurgical interventions. However, due to typical wavelengths in the millimeter range, it cannot sufficiently resolve microrobots. Therefore, US should be combined with different contrast-enhancing agents, exploiting the non-linear acoustical properties of microbubbles for contrast-enhanced US (CEUS)^52^, optical adsorption for multispectral optoacoustic tomography (MSOT) and PAI, a different movement reacting to a magnetic field for magneto-motive US (MMUS), or active beacons with coded responses^53^.

Remote-controlled microsystems need to be externally provided with propulsion and guidance toward a target with sufficient force/momentum. A common approach is to have magnetic microrobots that react to the field of external permanent magnets or electromagnets. A torque exerted from the magnetic field can change the orientation of magnetic microrobots and catheters for steering. Furthermore, a forward motion can be generated by rotating helical-shaped objects. Another mechanism for propulsion is gradient pulling, where a magnetic field gradient can exert a direct force on magnetic microrobots. Typically, the applied magnetic field strength is in the order of a few mT, which is almost three orders of magnitude less than that in clinical magnetic resonance imaging (MRI) devices. Exposure to magnetic fields of this strength is considered safe in general, even prenatal exposures to a magnetic field of 1.5 T during the second and third trimester of pregnancy in a cohort of 72 healthy fetuses showed no adverse effects on birth weight, long-term neurodevelopmental outcomes, growth, motor functioning, social or neurological development^54,55^.

Scaling up experiments with magnetic actuation from small animals, such as mice, to human scale, can require a penetration depth increased by one order of magnitude. As the magnetic field strength decreases proportionally to the cube of the distance, the magnetic field generation has to be increased by a factor of 100. In the case of electromagnets, this requires a 100-fold increase in electrical current times the number of windings, with an accompanying rise in mechanical and thermal load. The gradient of a magnetic field decays even faster, with the fourth power of the distance^56^, thus requiring a 1000-fold higher field generation. Magnetic actuation for human-scale applications will mostly be based on the transfer of torque, as the scaling laws are more favorably compared to gradient-based propulsion. Magnetic actuation systems are becoming more and more commercially available, such as the Navion (MagnebotiX, Zurich, and Switzerland), and are aiming for approval as medical devices^57^.

A recent approach for providing external propulsion to microrobots in vivo is based on the mechanical forces exerted by an ultrasound beam, based for instance on the acoustical streaming effect^58^, acoustical trapping, or the excitation of bubbles in a microswimmer^59^. Due to the ability to collimate or focus an ultrasound beam, the decay of the amplitude over the distance is mostly determined by the attenuation of the material. If the penetration depth should be increased from 10 to 100 mm for a collimated ultrasound beam through a typical biological tissue with an attenuation coefficient of α = 1 dBcm^−1^ MHz^−1^ at 1 MHz^60^, the output amplitude only has to be increased eightfold. This favorable scaling law together with fast beam steering and the possibility to combine imaging and actuation make ultrasound-based actuation a promising candidate for microrobotic interventions. Furthermore, the ability to have multiple independent beams enables multi-agent control even with several identical microrobots.

Complex organ systems with intertwined luminal and branching, such as the female reproductive tract, require advanced planning of the trajectories for remote-controlled microrobots. This can be either solely based on real-time imaging or include pre-operative imaging modalities. In the latter case, a registration of the pre-operative data with the live data that is robust to perturbations like physiological movements, such as breathing, is required. The planned trajectories can be manually defined by the surgeon and augmented with automated path proposals or fully automatic based on machine learning^61^.

## Ethical and regulatory considerations for a clinical application

The development of microrobots toward clinical applications in reproductive medicine requires addressing several distinct ethical and regulatory aspects:

i. The potential use of novel technology/medical devices in reproductive medicine and gynecology has to be balanced against potential risks/advantages and existing alternative approaches. More delicately, the potential targeting of biomaterial containing cellular constructs towards germ cells may interfere with the strict embryonic protection laws in place in several countries (e.g., Germany, Federal Law Gazette, Part I, No. 69, issued in Bonn, 19 December 1990, page 2746). In such cases, ex vivo applications may be advisable.
ii. Ethical commissions might be more willing to discuss the clinical use of such novel and complex therapeutic approaches in life-threatening diseases like cancer or other injuries in the reproductive system. In such indications, an ethical board approval may be willing to consider e.g., the targeted delivery of compounds towards malignant cells/undesired tissue growing.
iii. Again, most authorities would classify the non-biological part of a microrobot as a “medical device” and would follow the respective regulatory pathway (e.g., EU directive for medical devices)^62^. The lowest risk category I, accordingly would apply to devices that can be used with no or very low risk for a human being (e.g., diagnostic testing). All applications in which microrobots would be injected into body openings or fluid would be categorized II or III with consecutively higher hurdles for licensure.
iv. The combination of a medical device with a pharmaceutical compound or a living cell would be seen as a ‘combined’ product from the regulatory perspective. This results in a more complex approval procedure as existing knowledge regarding the bio-distribution, preclinical safety and toxicity for each separate component can not be used directly. Thus, a full risk-benefit assessments has to be conducted for novel combined products.
v. It can be assumed that using this technology (considered minimally invasive due to the small size of medical microrobots), the use of general anesthesia is not required.
vi. The possible administration route of microrobots into the human body in the case of embryo/gamete transfer could be in analogy to the vaginally performed and possibly ultrasound-guided embryo transfer or artificial insemination.
vii. The microrobots must fulfill existing standards including sterility comparable to IVF culture media used for embryo transfer. In analogy to IVF, culture media studies have to be performed before introducing them to humans.
viii. International regulatory authorities like FDA or EMA have started developing position statements and regulatory frameworks trying to address the increasing interest in the application of therapeutics containing nanomaterials (FDA Nanotechnology Task Force, “Nanotechnology Task Force Report 2007,” at ii (July 25, 2007))^63^, which should be also taken into consideration of the proposed microrobots/carriers decorated with nanomaterials for example for enhanced imaging contrast or combined therapies.

Keeping the aforementioned challenges in mind, it seems highly advisable to involve national and international competent regulatory authorities (e.g., Paul-Ehrlich Institute, EMA, FDA) as early as possible throughout the translational development of a micro/nanobot application. Several national authorities offer “scientific advice” to researchers, to determine prerequisites for preclinical safety testing (e.g., large animal data, stability, tumorigenicity, etc.). More recently, this advice can even be obtained from several national authorities in parallel^64^. Together with clinical research organizations (CRO), this will allow academic organizations or start-up companies to save costs when developing and applying for “first-in-human” microrobot applications.

## Summary

Reproductive medicine is still an evolving and modern field of medicine. The unmet need for more successful embryo transfers with subsequent implantation and pregnancy has to be addressed. The targeted transport of an embryo to the endometrium by microrobots may be an interesting approach to increase implantation rates in recurrent implantation failure cases. Another application of microrobots in assisted reproduction is the transport of gametes to the fallopian tube with its physiological culturing properties for the developing embryo. The microrobotic intratubal transfer offers the chance to reduce the time of ex vivo culture in IVF treatments and possible oxidative stress caused by human manipulation during the washing and incubation steps.

The discussed microrobotic ZIFT/GIFT will not replace the currently well-established treatment in ART but could represent an alternative solution in the future for minimally or noninvasive in vivo medical operations in general, and in the field of reproductive medicine. We believe it will be promising to perform a great part of the fertilization and embryo development process under more physiological conditions, which are difficult to recreate in vitro, reducing the oxidative stress on the gametes and preparing them for either in vivo fertilization or early embryo transfer for a synchronized and prepared implantation, and can be extended as mentioned before to other organs, and medical applications. The use of microrobots may improve the well-being of the patient by replacing an invasive surgery, which induces stress and requires additional anesthesia. We consider this especially relevant, because RIF patients may already suffer from increased anxiety and psychological distress.

Diseases in the reproductive system, like gynecological cancers, endometriosis, and tubal blockage, among others, will also benefit from this technology and are envisioned applications from our group. In general, the microrobotics field is quite new, especially in the scope of medical applications. There are just a few studies done in small animals like mice, and the results obtained from those studies showed evidence that microrobots are more efficient than passive drug administration carriers, as they have controllable motion and function. Additionally, they can be modified with nanomaterials and smart coatings so that they can release other cargoes (i.e., drugs) on demand and in a targeted fashion. In particular, our group has already demonstrated the use of drug-loaded sperm cells to treat tumor spheroids in vitro for cervical and ovarian cancer, outperforming conventional drug administration methods^47,65,66^. Recently we reported on multifunctional carriers for multiple sperm transport, local sperm capacitation, and release of hyaluronidase to assist the removal of cumulus cells in situ^28^.

Nevertheless, several specific considerations for using microrobots in reproductive medicine in animals, especially those in extinction and humans must be addressed in the future and discussed with patients, advocates, and regulators. In the meantime, applying microrobots to life-threatening diseases like cancer might be envisioned to obtain earlier information on the tolerability and safety of tethered and untethered microrobot applications in first-in-human clinical trials. Such experience might pave the way for the specific application outlined herein.
